# Possible Association of Nucleobindin-1 Protein with Depressive Disorder in Patients with HIV Infection

**DOI:** 10.3390/brainsci12091151

**Published:** 2022-08-29

**Authors:** Yun Yang, Qian Zhang, Jing Yang, Yun Wang, Ke Zhuang, Changcheng Zhao

**Affiliations:** 1 The First Affiliated Hospital of USTC, Division of Life Sciences and Medicine, University of Science and Technology of China, Hefei 230022, China; 2 ABSL-III Laboratory at the Center for Animal Experiment, State Key Laboratory of Virology, Wuhan University, Wuhan 430071, China

**Keywords:** depression, anxiety, mental disorders, nucleobindin 1, cannabinoid receptor 1, temporal cortex, neuron, cerebrospinal fluid, human immunodefciency virus, people living with HIV

## Abstract

Background: Mental disorders linked with dysfunction in the temporal cortex, such as anxiety and depression, can increase the morbidity and mortality of people living with HIV (PLWHA). Expressions of both nucleobindin 1 (NUCB1) and cannabinoid receptor 1 (CNR1) in the neurons have been found to alter in patients with depressive disorder, but whether it is involved in the development of depression in the context of HIV infection is unknown. Objectives To investigate the effects of NUCB1 on depressive disorder among PLWHA and preliminarily explore the underlying molecular mechanisms. Methods: Individuals who were newly HIV diagnosed were assessed on the Hospital Anxiety and Depression scale (HADS). Then SHIV-infected rhesus monkeys were used to investigate the possible involvement of the NUCB1 and the CNR1 protein in depression-like behavior. Results: The prevalence rate of depression among PLWHA was 27.33% (41/150). The mechanism results showing elevated NUCB1 levels in cerebrospinal fluid from HIV-infected patients suffering from depression were confirmed compared to those of HIV-infected patients. Moreover, the immunohistochemical analysis indicated the expression of NUCB1 in the temporal cortex neurons of SHIV-infected monkeys was higher than that of the healthy control. Conversely, CNR1 expression was down-regulated at protein levels. Conclusions: Depression symptoms are common among PLWHA and associate with NUCB1 expression increases, and NUCB1 may be a potential target for depression.

## 1. Introduction

Human immunodefciency virus (HIV) is one of the major public health problems that had affected over 37 million people worldwide by the end of 2020 [1]. People living with HIV (PLWH) present a series of mental disorders, such as depression and anxiety [2,3]. Studies have shown that the prevalence and complexity of depression is higher among PLWH than in the general population, as depression can be a risk factor for HIV acquisition as well as a consequence of HIV infection [4,5]. In recent years, research has explored possible mechanisms underlying depression among PLWHA, thereby discovering new diagnostic markers and novel therapeutic targets, becoming hotspots in the field of both AIDS and neurobiology research.

Nucleobindin 1 (NUCB1), also known as CALNUC or NUC, is a highly conserved, multifunctional protein widely expressed in nerve cells [6]. Importantly, it is believed to be a novel pan-neuronal calcium handling marker [7], associated with cancers [8], and stimulating insulin secretion [9]. According to the literature, NUCB1 protein is implicated in Alzheimer’s disease [10,11], other neurodegenerative diseases, or cellular processes known to be dysregulated early in tauopathy pathogenesis [12] such as synaptic plasticity, proteostasis, glucose metabolism, and mitochondrial function. Recently, a paper indicated that an adult-viable mutant that completely disrupts the G protein α-subunit binding and activates NUCB1 plays a neuroprotective role in the *Drosophila* model [13] of neurodegenerative disease. Furthermore, in 2020, an LC-MS/MS analysis predicted a significantly negative correlation between plasma protein levels of NUCB1 and degree of depression [14]. Therefore, we put forward the hypotheses that alterations in NUCB1 expression might be linked to depression among PLWHA.

It has been reported that depressive disorder is related to temporal lobe dysfunction [15,16], and bilateral thinning within the temporal cortex in HIV patients has been detected [17,18]. The present study aimed to survey the prevalence of depression among PLWHA and preliminarily explore the underlying mechanisms. However, brain tissues from PLWH are not readily available, thereby limiting research access to mechanisms underlying depression in the context of HIV infection. Considering simian immunodeficiency virus (SIV) or simian/human immunodeficiency virus-infected (SHIV) rhesus macaques (RMs) have been widely utilized in pathogenic mechanisms of neuroAIDS [19,20] to unravel the possible involvement of NUCB1 in the pathophysiology of depression among PLWHA, we studied SHIV-infected RMs because the viral replication and pathological changes in the brain of infected RMs resembles those in PLWHAs. Understanding the mechanism underlying the interaction between NUCB1 expression and depression in the context of HIV-infection may provide insights that may facilitate the development of a biomarker for diagnosis, new drug target, and treatment response.

## 2. Material and Methods

### 2.1. Research Subjects

In order to eliminate the potential effects of other factors, from January 2015 to October 2020, a total of 150 individuals were selected at the First Affiliated Hospital of the University of Science and Technology of China (USTC). Eligibility criteria inclusion for participation in the present study were newly diagnosed as HIV positive, no current prescription for antiretroviral medication, and having given informed consent/assent to participate in the study. The exclusion criteria were a diagnosis of schizophrenia or other psychotic disorder, bipolar disorder, substance dependence, dementia, or other neurodegenerative disease that could significantly impact cognitive functioning, or a mood disorder due to a general medical condition or substance use. All participants were asked to participate in the HADS questionnaires and the medical history systems. This study has been reviewed and approved by the Bioethics and Biological Safety Review Committee of USTC (2021-N(H)-236).

### 2.2. Investigation Procedures

HADS was used to assess severity of anxiety and depression symptoms, where A represents anxiety, while D represents depression [21,22]. The score of each subscale ranges between 0 and 21 points. Overall, the total scores of HAD-A and HAD-D were classified as normal (0–7) and anxiety or depressive (8–21), with higher scores indicating higher levels of symptoms [23].

### 2.3. Western Blot Assay

Night cerebrospinal fluid (CSF) samples (HAD-D score ≥ 16) were obtained by lumbar puncture in the morning; 3 mL CSF was taken from each subject. Western blot was performed as previously described [24]. Briefly, equal amounts of proteins from the CSF samples (40 microg/each lane) were separated by SDS-PAGE gel and electrophoresed, then transferred to a nitrocellulose membrane followed by blocking with 5% non-fat milk for 1 h and incubated with primary antibody (anti-NUCB1 Rabbit pAb, ABclonal, A3994) at 4 °C overnight. Appropriate secondary antibodies (1:2000, Santa Cruz, CA, USA) were used for two-hour incubation at room temperature. Membranes were visualized by ECL plus kit (GE Healthcare and Life Science, Piscataway, NJ, USA).

### 2.4. Animals and Ethics Statement

The RMs used in this study were from the Tianqin Breeding and Research Center (SCXK (e) 2021–0010), Hubei Province, China. The monkeys were housed in an air-conditioned room with an ambient temperature of 16–26 °C, a relative humidity of 40–70%, and a 12 h light-dark cycle at the Animal Bio-Safety Level-III laboratory of the Wuhan University (SCXK (e) 2019–0013). The RMs were individually housed in stainless steel wire-bottomed cages with sufficient space (800 mm wide, 800 mm depth, and 1600 mm height) and provided with a commercial monkey diet. In addition, animal health was monitored daily by the animal care staff and veterinary personnel. All study protocols were approved by the Institutional Animal Care and Use Committee of USTC (2021-N(A) -349) in accordance with the regulations of the National Institute of Health “Guide for the Care and Use of Laboratory Animals”, and all details of animal welfare and steps taken to ameliorate suffering were in accordance with the recommendations of the Weatherall report, “The use of nonhuman primates in research”.

### 2.5. Virus Inoculation and Sample Collection

Seven Chinese-origin RMs from three different projects were enrolled in this study. Of these, five RMs were inoculated with 10^3^–10^8^ TCID_50_ of SIV/SHIV by either intravenous or intravaginal route under anesthesia with intramuscular injection of ketamine hydrochloride (10 mg/kg) plus intramuscular injection of atropine (0.04 mg/kg), while two healthy RMs were inoculated with the same volume of medium for mock infection and were used as negative controls.

### 2.6. Histology and Immunohistochemistry

At day of sacrifice, a depressive monkey and a healthy control were anesthetized with ketamine-HCl and euthanized by intravenous pentobarbital overdose. Formalin-fixed, paraffin-embedded brain sections from the cerebrum were obtained; 4 microns sections were processed and stained with hematoxylin and eosin (H&E) staining and immunohistochemistry (IHC). For IHC, sections were secured using an automated system, the Dako Autostainer Link. Formalin-fixed paraffin sections were rehydrated with water. Heat-induced epitope retrieval was performed with the FLEX TRS High-pH Retrieval Buffer for 20 min. After peroxidase blocking, the specific monoclonal antibody (IHC-plus CNR1/CB1 pAb, Lifespan, LS-B8253; anti-NUCB1 Rabbit pAb, ABclonal, A3994) was applied at room temperature for 20 min. The FLEX + Rabbit EnVision System was used for detection. DAB chromogen was then applied for 10 min. Slides were counterstained with Mayers hematoxylin for 5 s and then dehydrated and coverslipped. Images were then processed and analyzed using CaseViewer software (2.1 v, 3D Histech Ltd., Budapest, Hungary). Negative controls were included in the run.

### 2.7. Western Blotting

Western blotting was performed in cerebral cortex tissue lysates as previously described [25]. Briefly, tissue lysates of the cerebral cortex from SHIV-infected RMs were lysed with radioimmune precipitation assay buffer. Subsequently, proteins were transferred onto nitrocellulose membranes (Bio-Rad, Hercules, CA, USA), and appropriate primary antibodies and HRP-conjugated secondary antibodies were used. Proteins were detected with the enhanced chemiluminescent (ECL) reagent (Thermo Scientific, Waltham, MA, USA), followed by quantification using ImageJ software.

### 2.8. Statistical Analysis

Statistical analysis of the data was performed using the chi-square test (level of significance was 0.05) with SPSS software version 23.0 (Chicago, IL, USA) and GraphPad prism 5.0 (GraphPad Software, San Diego, CA, USA). In each aspect, univariate analysis was used to determine the variables significantly related to the dependent variable. The confidence interval was 95%.

## 3. Results

### 3.1. Prevalence of Depression among HIV-Infected Patients

Amongst all study participants, 6.00% (9/150) of patients newly testing positive for HIV met the criteria for depression (HAD-D score ≥ 8), and the prevalence of baseline anxiety was 14.00% (21/150) according to the HAD-A score ≥ 8 criterion. Combined anxiety and depression accounted for 21.33% (32/150) of the variance in reported body dissatisfaction. Furthermore, the number of HIV-infected individuals with HAD-A score ≥ 8 was reduced significantly from 53 at baseline to 27 at week 8 (*p* = 0.006), but no statistically significant differences in the number of patients with HAD-D score ≥ 8 was observed comparing baseline to week 8 (*p* = 0.189, two sample *t*-test). Prevalence of anxiety and depression among PLWHAs are listed in Table 1, respectively.

### 3.2. Univariate Analyses of Variables Related to Symptoms of Depression among Individuals with HIV Infection

We included personal information and clinical information in univariate analyses to observe whether the variables were related to anxiety and depressive symptoms. A total of 150 participants were included in the study. Nearly all participants were male (146/150; 97.33%). The mean age and weight of the study participants was 34.87 (± standard deviation 14.15) years and 67.02 ± 11.91 kg, respectively. Participants were mostly living without a spouse (118/150; 78.67%), were private employees (96/150; 64.0%), and had some education (104/150; 69.33%). Most of the study participants were homosexual. The time elapsed from the first positive HIV test to ART initiation of over half (108/150; 72.0%) was less than 4 weeks. Moreover, 28 (18.7%) had comorbid smoking, and drinking alcohol (16.0%). Demographic characteristics are shown in Table 2. Among the several items, “age” and “marital status” were significantly related to depression in those patients using univariate analyses (both *p* < 0.05). In addition, “weight” was significantly related to the patient’s anxiety symptom (*p* = 0.043) among the variables.

### 3.3. NUCB1 Levels Are Elevated in CSF from HIV Cases Currently Suffering from Depression

**Figure 1:**
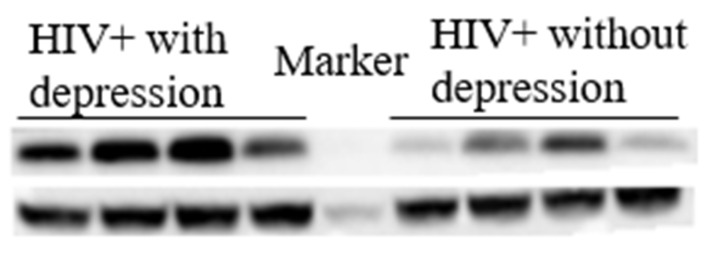
Representative image of protein levels of NUCB1. Western blotting results showed that in HIV-infected individuals, having comorbid depression significantly increased the expression of NUCB1 in the cerebrospinal fluid.

CSF is a proximal fluid which communicates closely with brain tissue and contains numerous brain-derived proteins. Thus, NUCB1 expressions of CSF in HIV-infected individuals were examined via Western blot analysis. Comparing with only HIV cases, the protein expression of NUCB1 in the CSF was significantly increased in those HIV cases currently suffering from depression. Notably, we did not enroll a control group of healthy subjects because of the invasiveness of the CSF procedure for which the Ethics Committee did not allow. Protein levels of NUCB1 in CSF are reported in Figure 1.

### 3.4. SHIVKU-1 Infection Triggers a Reduction in the Number of Neurons in Cerebral Cortex of Rhesus Monkeys

To explore the pathogenesis of depression among PLWHA, in this study, SIV/SHIV of five RMs established persistent infections. Of note, during the late stage of infection, Macaque WSH01 infected with SHIV_KU-1_ presented depression-like syndromes [26,27] that mimic those observed in human neuroAIDS, including difficulties in standing, head tilting, weakening of muscle strength, decreased appetite and movement, loss of body mass, ataxia, fear, psychomotor changes, sleep disturbance, and total loss of motoric function on the left side of the body. The animal had a slow progressing course lasting for about 18 months after the triggering infection and was euthanized at week 72. This study demonstrated that SHIV_KU-1_-infected RMs can resemble human neuroAIDS and will become an important tool for studying pathogenesis and evaluating treatment and preventive drugs of neuroAIDS.

**Figure 2:**
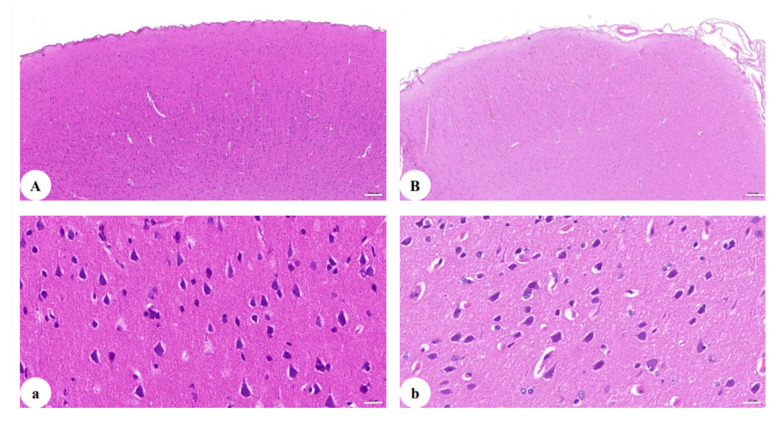
H&E staining of the cerebral cortex: (**A**) section of the cerebral cortex of healthy monkey (50×); (**B**) section of the cerebral cortex of the SHIV_KU-1_-infected monkey brain (50×); (**a**) section of the cerebral cortex of healthy monkey (400×); (**b**) section of the cerebral cortex of the SHIV_KU-1_-infected monkey brain (400×).

H&E staining analysis (Figure 2A,B) showed marked histological damages with increased infiltration and vasculitis in the brain of Macaque WSH01. Importantly, the cerebral cortex exhibited a decrease in the number of neurons. Accompanied with purulent meningitis, the intactness of the cerebral cortex could be not revealed more clearly, and many cavities appeared after liquefaction of cerebral cortex tissue. Specifically, the neuronal loss in the cerebral cortex of the SHIV_KU-1_-infected monkey was observed, and there were also substantial changes in the spatial arrangement of neurons. In addition, residual nerve cells bodies in the cerebral cortex changed from swelling to shrinkage, Nissl body staining was weak, the dendritic length due to cell death or an inflammatory process was irregular, and neural axone was thin (Figure 2a,b).

### 3.5. Up-Regulation of NUCB1 Protein in Cerebral Cortex of SHIV_KU-1_-Infected Monkey

**Figure 3:**
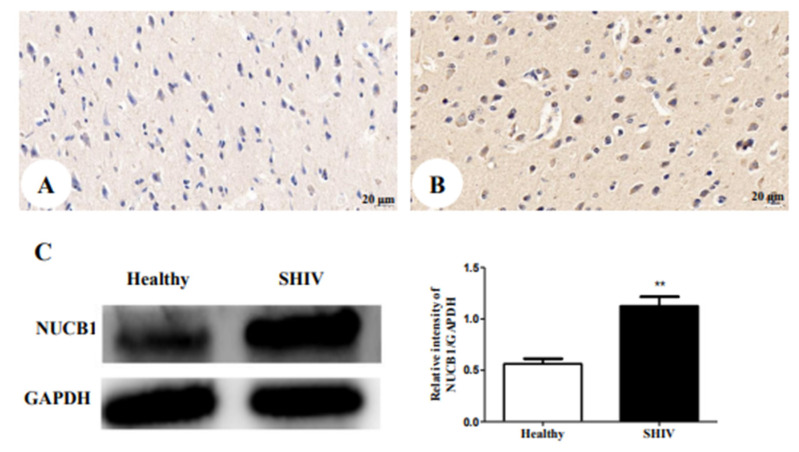
NUCB1 protein expression in cerebral cortex: (**A**) the healthy control (400×); (**B**) SHIV_KU-1_-infected monkey (400×) (**C**) comparison of NUCB1 expression of cerebral cortex in healthy control and SHIV_KU-1_-infected monkey using Western blotting. In the column, from the *t* test, statistically significant differences of the two groups can be determined (*p* = 0.0056). ** *p* < 0.01.

We next verified whether alterations in the protein expression of NUCB1 occurred in an SHIV_KU-1_-infected monkey via IHC staining analysis (Figure 3A,B). The results revealed that the NUCB1 protein was expressed in the cerebral cortex of the healthy monkey but, in the infected one, the expression of the NUCB1 protein was higher. Furthermore, these findings were supported by data from quantitative Western blotting of NUCB1 protein levels in the whole cerebrum in vivo (*p* = 0.0056) (Figure 3C). The results suggest that NUCB1 plays an important role in the pathological processes leading to depression.

### 3.6. Down-Regulation of CNR1 Protein in Cerebral Cortex of SHIV_KU-1_-Infected Monkey

Finally, 16 potential targets relative to depression were acquired from the TCMSP database (https://old.tcmsp-e.com/disease.php?qd=228, accessed on 19 March 2022) and further analyzed by the online STRING database (http://string.embl.de/, accessed on 15 March 2022) to explore the functional mechanism of the NUCB1 protein.

**Figure 4:**
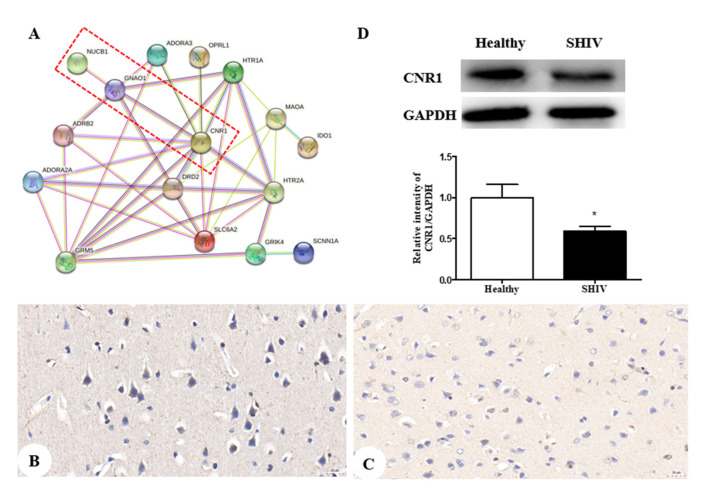
CNR1 protein expression in both an SHIV_KU-1_-infected monkey and healthy control: (**A**) the protein-protein interactome networks; blue rectangle nodes represent down-regulated proteins; red circular nodes stand for the up-regulated proteins; the lines represent the regulation of relationship between two nodes; (**B**) IHC staining against CNR1 of the healthy control (400×); (**C**) IHC staining against CNR1 of the SHIV_KU-1_-infected monkey (400×); (**D**) comparison of CNR1 expression of cerebral cortex in healthy control and the SHIV_KU-1_-infected monkey using Western blotting. In the column, from the *t* test, statistically significant differences of two groups can be determined (*p* = 0.0366). * *p* < 0.05.

The network showed CNR1, neuroprotective and highly expressed in the neurons [28,29], is in close contact with NUCB1 (Figure 4A). Thereby, we investigated the impact of SHIV_KU-1_ infection on CNR1 expression in neurons. Accordingly, we observed, using an immunohistochemical technique, the down-regulation of CNR1 protein expression in the infected monkey compared to the healthy control (Figure 4B,C). This was supported by protein levels of CNR1 via Western blot analysis (*p* = 0.0366) (Figure 4D). We speculate that decreased CNR1 expression by SHIV_KU-1_ infection results in depressive disorder because there is support for existing cannabinoid signaling pathways that can decrease neuronal injury [30].

## 4. Discussion

Many studies have reported significantly higher prevalence of depression in HIV-infected patients when compared to the general population [31,32]. In the present study, the prevalence of depression among HIV-infected patients was 27.33% (41/150). Moreover, univariable logistic regression analysis indicated that age and marital status were associated with depression (*p* < 0.05). Unfortunately, the current diagnoses of depression are still based on clinical manifestations and self-rating scales as the main diagnostic criteria, as a lack of relevant objective laboratory indicators exists. Thus, there is an urgent need to search for and identify new clinical biomarkers of depression.

Increased functional connectivity between the temporal lobe dysfunction and the DMN (default mode network) has been shown in depressive disorder [33,34]. Evidence from recent studies provides a deeper understanding of human learning neural networks, particularly on human PFC crucial role, proposing a theoretical model to conceptualize these psychophysiological processes. The neurovisceral integration model of fear (NVI-f) that can be impaired in the context of a psychiatric disorder [35,36] might also contribute to the advancement of alternative, more precise and individualized treatments for psychiatric disorders. Moreover, recent studies demonstrated regulatory and functional aspects of the kynurenine pathway, which has been shown to possess neuroprotective and antidepressant-like properties [37,38]. In addition, there is evidence [39,40] that diet may also protect from depressive disorders and improve mental health through mechanisms that are related to inflammation, endogenous metabolic factors in cognitive and emotional functions, and pathological neural substrates of depressive disorder, especially on frontal lobe dysfunction [41].

Fortunately, our understanding of the (endocannabinoid system, ECS) comprised neuromodulatory lipids and their receptors associated with depression has increased [42,43]. CNR1, also known as CB1, is the most abundant G protein-coupled receptor (GPCR) in the mammalian brain. Rocha et al. [44] found that CNR1 knockout mice lost weight and appetite, reduced rearing and exploratory behaviors, and increased anxiety, compared to wild-type littermates. These symptoms were similar to human depression patients. In addition, a clinical phase I/II trial with SR14716A (rimonabant), a CNR1 antagonist agonist showed that it produced serious adverse neuropsychiatric events such as anxiety, depression, and even suicidal ideation [45,46].

**Figure 5:**
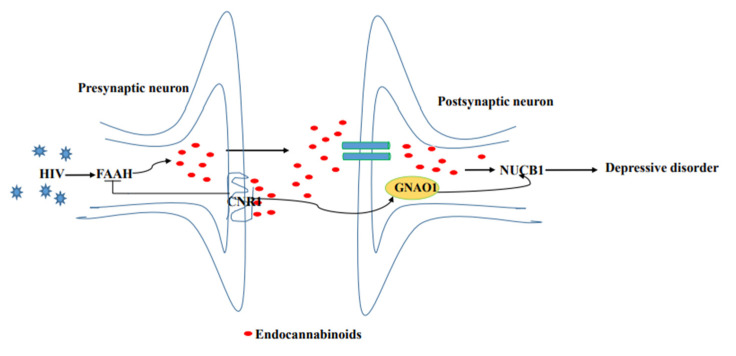
Proposed model of the molecular events in NUCB1-mediated HIV comorbid depression.

Several studies have showed that HIV gp120 stimulates increased cortical fatty acid amide hydrolase (FAAH) [47,48], subsequently allowing for rapid 2-AG and AEA production [49], selective ligands for the NUCB1 linked to degree of depressive behavior [50], in the postsynaptic neuron, whereas decreased CNR1 expression promotes the release of 2-AG and AEA [51,52]. Herein, down-regulation of CNR1 expression and up-regulation of NUCB1 expression in neurons were found in the co-occurrence of depression disorder and SHIV infection, as detected by IHC and Western blot. By combining our experimental results, we propose a schematic presentation of a possible mechanism of NUCB1 involvement in depression in PLWH (Figure 5). Therefore, NUCB1 inhibition may be considered as a therapeutic agent to relieve depressive disorder. Based on the published articles, we speculate that both a ZiBuPiYin recipe [53], which was recorded in the book of Bujuji written by Cheng Wu in the Qing dynasty, and Luks-PV [54] which is a pore-forming leukocidin secreted by *Staphylococcus aureus*, have potential roles in the prevention and treatment of depression because of their reductions in the levels of the NUCB1 protein.

There are several limitations in our study. Firstly, with an animal model we were unable to conduct questionnaires, interviews, or oral reports. Therefore, the severity of depression cannot be quantified. Moreover, there is no way for a human investigator to really know whether a monkey is feeling depressed. What we can do is observe the behavior that a monkey makes in response to viral infection. Second, there are too little data on NUCB1 levels in CSF from HIV-infected individuals currently suffering from depression to draw a convincing conclusion. The sample size should be enlarged, etc. Third, a total of 150 participants were randomly selected rather than by performing a statistical power analysis to estimate the appropriate number of participants required to generate results. Finally, further studies are needed to demonstrate the expression of NUCB1 in specific neuronal subpopulations. Despite all this, this study suggests that the NUCB1 protein may be a novel clinical biomarker for depression, and inhibiting its activity might have a potential function that predicts and monitors responsiveness of treatment. In future studies, we will investigate the molecular mechanisms underlying the regulation of NUCB1 expression and the role of NUCB1 in other mental disorders.

Overall, this study indicates a high prevalence of depressive symptoms in newly diagnosed HIV-infected patients and identifies NUCB1-CNR1 signaling as a novel mediator of impaired neuronal function in HIV-infected people. Together, these findings illustrate that NUCB1 inhibition can provide an additional neuroprotective benefit, extending the significance of our findings beyond HIV.

## Figures and Tables

**Table 1:** Prevalence of anxiety and depression among HIV/AIDS patients.

Items	HAD-A		HAD-D	
	Week 0	Week 8	Week 0	Week 8
	≤7	≥8	≤7	≥8
Score ≤ 7	97	105	109	105
Score ≥ 8	53	27	41	27
x^2^		7.443		1.723
*p*		0.006		0.189

**Table 2:** Factors associated with anxiety and depression among patients with HIV/AIDS.

Factors	n	Anxiety (HAD-A ≥ 8)	*Χ* ^2^ *p*		Depression (HAD-D ≥ 8)	*Χ* ^2^ *p*	
Age			0.909	0.464		5.419	0.001 *
≤20 years	20	10			4		
21–30 years	64	24			14		
31–40 years	38	12			10		
41–50 years	14	6			6		
≥51 years	14	8			10		
Gender			0.025	0.876		0.423	0.517
male	146	60			43		
female	4	1			1		
Marital status			0.039	0.962		3.270	0.044 *
Single	96	38			22		
married	32	16			12		
divorced/widowed	22	10			10		
Weight (kg)			3.296	0.043 *		0.735	0.483
≤60	52	28			16		
61–80	84	28			28		
≥81	14	6			0		
Education			2.469	0.069		2.135	0.103
unable to read and write or primary	6	6			4		
secondary	40	20			14		
junior college	52	18			14		
diploma and above	52	18			12		
Occupation			1.386	0.257		0.610	0.546
permanent employee	32	16			12		
private employee	96	34			28		
student	22	10			4		
HIV transmission route			0.029	0.865		0.733	0.395
homosexual	126	52			38		
heterosexual	24	8			6		
Time elapsed from the first positive HIV test to ART initiation			0.054	0.947		0.799	0.454
≤4 week	108	44			28		
week 4–8	20	8			8		
≥8 week	22	8			8		
Smoking			0.010 *	0.991		0.170	0.844
never	122	50			36		
≤10 cigarettes per day	20	8			6		
≥10 cigarettes per day	8	2			2		
Drinking			0.871	0.424		0.748	0.478
never	126	52			30		
occasionally	20	6			6		
≥100mL liquor per day	4	2			4		

Note: * significant difference.

## Data Availability

The original contributions presented in the study are included in the article, further inquiries can be directed to the corresponding authors.
